# Enhancement of HGF-induced tubulogenesis by endothelial cell-derived GDNF

**DOI:** 10.1371/journal.pone.0212991

**Published:** 2019-03-07

**Authors:** Masao Nakasatomi, Shunsuke Takahashi, Toru Sakairi, Hidekazu Ikeuchi, Yoriaki Kaneko, Keiju Hiromura, Yoshihisa Nojima, Akito Maeshima

**Affiliations:** Department of Nephrology and Rheumatology, Gunma University Graduate School of Medicine, Maebashi, Japan; Center for Molecular Biotechnology, ITALY

## Abstract

Tubulogenesis, the organization of epithelial cells into tubular structures, is an essential step during renal organogenesis as well as during the regeneration process of renal tubules after injury. In the present study, endothelial cell-derived factors that modulate tubule formation were examined using an in vitro human tubulogenesis system. When human renal proximal tubular epithelial cells (RPTECs) were cultured in gels, tubular structures with lumens were induced in the presence of hepatocyte growth factor (HGF). Aquaporin 1 was localized in the apical membrane of these tubular structures, suggesting that these structures are morphologically equivalent to renal tubules in vivo. HGF-induced tubule formation was significantly enhanced when co-cultured with human umbilical vein endothelial cells (HUVECs) or in the presence of HUVEC-conditioned medium (HUVEC-CM). Co-culture with HUVECs did not induce tubular structures in the absence of HGF. A phospho-receptor tyrosine kinase array revealed that HUVEC-CM markedly enhanced phosphorylation of Ret, glial cell-derived neurotrophic factor (GDNF) receptor, in HGF-induced tubular structures compared to those without HUVEC-CM. HUVECs produced GDNF, and RPTECs expressed both Ret and GDNF family receptor alpha1 (co-receptor). HGF-induced tubule formation was significantly enhanced by addition of GDNF. Interestingly, not only HGF but also GDNF significantly induced phosphorylation of the HGF receptor, Met. These data indicate that endothelial cell-derived GDNF potentiates the tubulogenic properties of HGF and may play a critical role in the epithelial-endothelial crosstalk during renal tubulogenesis as well as tubular regeneration after injury.

## Introduction

Tubulogenesis is an essential process during renal organogenesis and during the repair process of renal tubules after injury. Many cellular events including cell growth, differentiation, apoptosis, proteolysis, and cytoskeletal organization are involved in this process[1, 2]. Tubulogenesis is regulated by interactions among different cell types. Various important environmental cues control tubulogenesis in vivo[3]. Given that endothelial cells form an extensive network of blood vessels that provide signals in a paracrine fashion to induce organ formation[4], endothelial-cell derived factors may regulate proliferation and differentiation of renal tubular cells during renal tubulogenesis as well as during the regeneration process of renal tubules after injury. However, analyzing the interaction between renal tubules and peritubular capillaries in vivo and identification of the soluble factor(s) regulating this interaction are difficult.

In vivo tubular regeneration involves the collaborative action of various growth factors and extracellular matrix components. This process can be mimicked by in vitro 3D tubulogenesis systems, which resemble the in vivo situation more closely than 2D cultures. This systems not only help to define common principles underlying the formation of diverse types of tubular organs[5], but also imitate the regeneration process of renal tubules after injury and are useful for understanding this process[6].

Hepatocyte growth factor (HGF) induces 3D tubular structures in Madin-Darby canine kidney (MDCK) cells cultured in collagen gels[7, 8]. The tubulogenic action of HGF can also be observed in other in vitro tubulogenesis assays using murine inner medullary collecting duct (IMCD)-3 cells[9–12], rat primary tubular epithelial cells[13], and human renal proximal tubular cells[14]. These culture systems are widely utilized for studying the growth factors and other signals involved in renal tubulogenesis as well as in the regeneration process of adult kidney after injury. Importantly, HGF has proven to be beneficial in animal models as well[15].

In the present study, we utilized an in vitro human tubulogenesis model to explore the endothelial cell-derived factors that regulate tubule formation during the regeneration process of renal tubules after injury. Human renal proximal tubular epithelial cells (RPTECs) cultured in gel formed tubular structures with lumens in the presence of HGF. HGF-induced tubule formation was significantly enhanced by co-culture with human umbilical vein endothelial cells (HUVECs). A phospho-receptor tyrosine kinase (RTK) array demonstrated that when cultured with HUVEC conditioned medium (HUVEC-CM), phosphorylation of Ret, glial cell-derived neurotrophic factor (GDNF) receptor, was markedly enhanced in HGF-induced tubular structures compared to those without HUVEC-CM. HUVECs produced GDNF, and RPTECs expressed both Ret and GDNF family receptor alpha1 (GFR-alpha 1; co-receptor). HGF-induced tubule formation was significantly enhanced by addition of GDNF. These data indicate that endothelial cell-derived GDNF potentiates the tubulogenic action of HGF in a paracrine manner. GDNF-Ret signaling may play an essential role in epithelial-endothelial crosstalk during tubule formation.

## Materials and methods

### Reagents

Human recombinant HGF (294-HGN-005) and GDNF (212-GD-010) were obtained from R&D Systems (Minneapolis, MN). Growth factor-reduced Matrigel (BD354230) was obtained from CORNING (Corning, NY), and atelocollagen (DME-02) was obtained from Koken (Tokyo, Japan). Antibodies used in this study were as follows: goat polyclonal anti-GDNF antibody (AF-212-NA) (R&D Systems); mouse anti-beta-actin antibody (3598R-100) (BioVision, Milpitas, CA); rabbit monoclonal anti-Ret antibody (E1N8X) (CST #14556) (Cell Signaling Technology, Danvers, MA); rabbit monoclonal anti-Ret antibody (ab134100) (Abcam, Cambridge, MA); anti-Met antibody (ab39075) (Abcam, Cambridge, MA); anti-phospho-Met antibody (CST #3077) (Cell Signaling Technology, Danvers, CO); anti-aquaporin 1 (AQP1) antibody (sc-20810) (Santa Cruz Biotechnology, Dallas, TX).

### Cell culture

Primary human RPTECs (Lonza, Walkersville, MD) were maintained in renal epithelial cell basal medium supplemented with REGM complex (hydrocortisone, human epidermal growth factor, epinephrine, triiodothyronine, transferrin, insulin, gentamicin sulfate, and 0.5% fetal bovine serum). Primary HUVECs (Clonetics, Walkersville, MD) were cultured in endothelial basal medium (HuMedia-EB2, Kurabo, Osaka, Japan) supplemented with HuMedia-EG (1.34 μg/ml hydrocortisone, 10 ng/ml human epidermal growth factor, 5 ng/ml human fibroblast growth factor-B, 10 μg/ml heparin, 50 μg/ml gentamicin, 50 ng/ml amphotericin B, and 2% fetal bovine serum). These cells were cultured in humidified conditions of 95% air/5% CO_2_ at 37°C. The culture medium of RPTECs was changed every 3–4 days. HUVEC culture medium was changed every 1–2 days. To collect HUVEC-conditioned media (HUVEC-CM), which contains the soluble factor(s) produced by HUVEC, confluent HUVECs were cultured in HuMedia-EB2 supplemented with HuMedia-EG for 48 h. Culture supernatant were collected and then centrifuged at 1500 rpm for 5 min at 4°C.

### Three-dimensional gel culture

RPTECs were suspended at 5 × 10^4^ cells/ml in a mixture of Matrigel and atelocollagen I (1:1). The cell solution was dispensed into 96-well culture plates and incubated at 37°C. After the solution had gelled, HuMedia-EB2 supplemented with HuMedia-EG was added. Cultures were photographed after 5 days using Nikon MFA10100 (Tokyo, Japan) equipped with OLYMPUS E-620 (Tokyo, Japan). To obtain the sections for histological analysis, gel cultures were fixed after 8–9 days in 10% formalin, paraffinized, and sectioned. Paraffin-embedded sections (4 μm) were cut and stained with periodic acid-Schiff (PAS).

### Quantitative analysis of tubule formation

RPTECs were cultured in gels with the indicated factors. After the indicated time periods, four high-power fields were randomly selected and digitized. The total length of tubular structures (μm) in each field was measured using image J (National Institutes of Health, Bethesda, MD). Values are the means ± standard error (SE) from 4 independent experiments with 4 replicates.

### Indirect fluorescence immunohistochemistry

Indirect immunofluorescence staining was performed as described previously [16]. Paraffin-embedded sections (4 μm) were deparaffinized with 100% xylene for 10 min twice, followed by hydration by soaking for 20 sec each in ethanol (100%, 90%, 80%, 70%, and 50%) and washed in sterile water. The sections were pretreated with 3% bovine serum albumin-phosphate-buffered saline (PBS) for 1 h, and incubated with primary antibody at room temperature for 1 h. After washing in PBS, sections were covered with a mixture of fluorescence-labeled secondary antibodies (Alexa 488 donkey anti-rabbit IgG) and 4’-diamidino-2-phenylindole (DAPI: Thermo Scientific, Rockford, IL). Immunofluorescence images were recorded with a Spot RT Slider digital camera attached to a Nikon Eclipse 80i fluorescence microscope. Rat kidney tissue was used as a positive control for AQP1 immunostaining.

### Western blot analysis

Cells were washed two times with cold PBS and suspended in RIPA lysis buffer (Santa Cruz Biotechnology, Dallas, TX). After centrifugation, supernatant was collected, and the protein concentration was determined with the Pierce^TM^ BCA protein assay kit (Thermo Scientific). Ten or fifteen micrograms of protein from each sample was separated by SDS-PAGE and transferred to a polyvinylidene difluoride membrane (Millipore, Bedford, MA). To reduce nonspecific antibody binding, the membrane was blocked with Tris-buffered saline (TBS: 20 mM Tris-HCl, 150 mM NaCl, and 0.1% Tween 20) containing 5% bovine serum albumin, incubated with primary antibody at 4°C overnight, and washed with TBS. After incubation with peroxidase-labeled secondary antibody for 1 h at room temperature, the membrane was washed with TBS and analyzed with ImageQuant LAS4000 (GE Healthcare, Buckinghamshire, UK) using ECL^TM^ Select Western Blotting Detection Reagent (GE Healthcare). Primary antibodies were used at 1:1000 dilution (anti-GDNF antibody), 1:2000 dilution (anti-Met antibody and anti-phospho-Met antibody) and 1:4000 dilution (anti-beta-actin antibody). Rat brain tissue was used as a positive control for GDNF expression.

### Protein arrays

RPTECs were cultured in gels with HuMedia-EB2 supplemented with HuMedia-EG or HUVEC-CM. The Human Phospho-RTK Array Kit (R&D Systems) was used according to the manufacturer’s instructions. Briefly, after 5 days, proteins were extracted from these gels with lysis buffer, which was a component of this kit, supplemented with 10 μg/ml Aprotinin (Sigma Aldrich, St. Louis, MO), 10 μg/ml Leupeptin, and 10 μg/ml Pepstatin (Tocris, Bristol, UK). After blocking, arrayed antibody membranes were incubated with 300 μg protein at 4°C overnight. After washing, membranes were incubated with horseradish peroxidase-conjugated antibodies for 2 h at room temperature and reacted with chemiluminescent substrate. The signal was detected with ImageQuant LAS4000 and quantified using ImageQuant TL (GE Healthcare). The signal intensities of target proteins were determined with subtraction of the negative control spot intensity. Data was shown as the relative expression normalized to the positive control spot intensity.

### Reverse Transcription-PCR (RT-PCR)

Total RNA was extracted from cells with the RNeasy Mini Kit (Qiagen, Hilden, Germany) according to the manufacturer’s instructions. First-strand cDNA was prepared using the Omniscript Reverse Transcription Kit (Qiagen). RT-PCR was performed with specific primers, the sequences[17–22] of which are shown in S1 Fig.

Reactions included 10× PCR buffer, MgCl_2_ (25 mM), dNTP mixture (2 mM), 3′ primer, 5′ primer, Taq polymerase (Thermo Scientific), and cDNA. Samples were incubated at 95°C for 5 min, followed by 35 cycles of 30 s at 95°C, 30 s at 58°C (GAPDH, GFR-alpha 1, GFR-alpha 2, GFR-alpha 3, GFR-alpha 4); 30 s at 54°C (NRTN, ARTN, PSPN); 5 s at 64°C (Ret), and 1 min at 72°C, with a final extension at 72°C for 5 min in a Veriti Thermal Cycler (Thermo Scientific). The sample for GDNF was incubated at 95°C for 5 min, followed by 35 cycles of 15 s at 95°C, 15 s at 58°C, and 1.5 min at 72°C, with a final extension at 72°C for 7 min in a Veriti Thermal Cycler.

### Statistical analysis

Statistical analysis were performed by two-tailed Student’s t-test for comparisons of two groups with SPSS (Chicago, IL). P values <0.05 were considered significant.

## Results

### Induction of tubular structures by HGF

**Fig 1:**
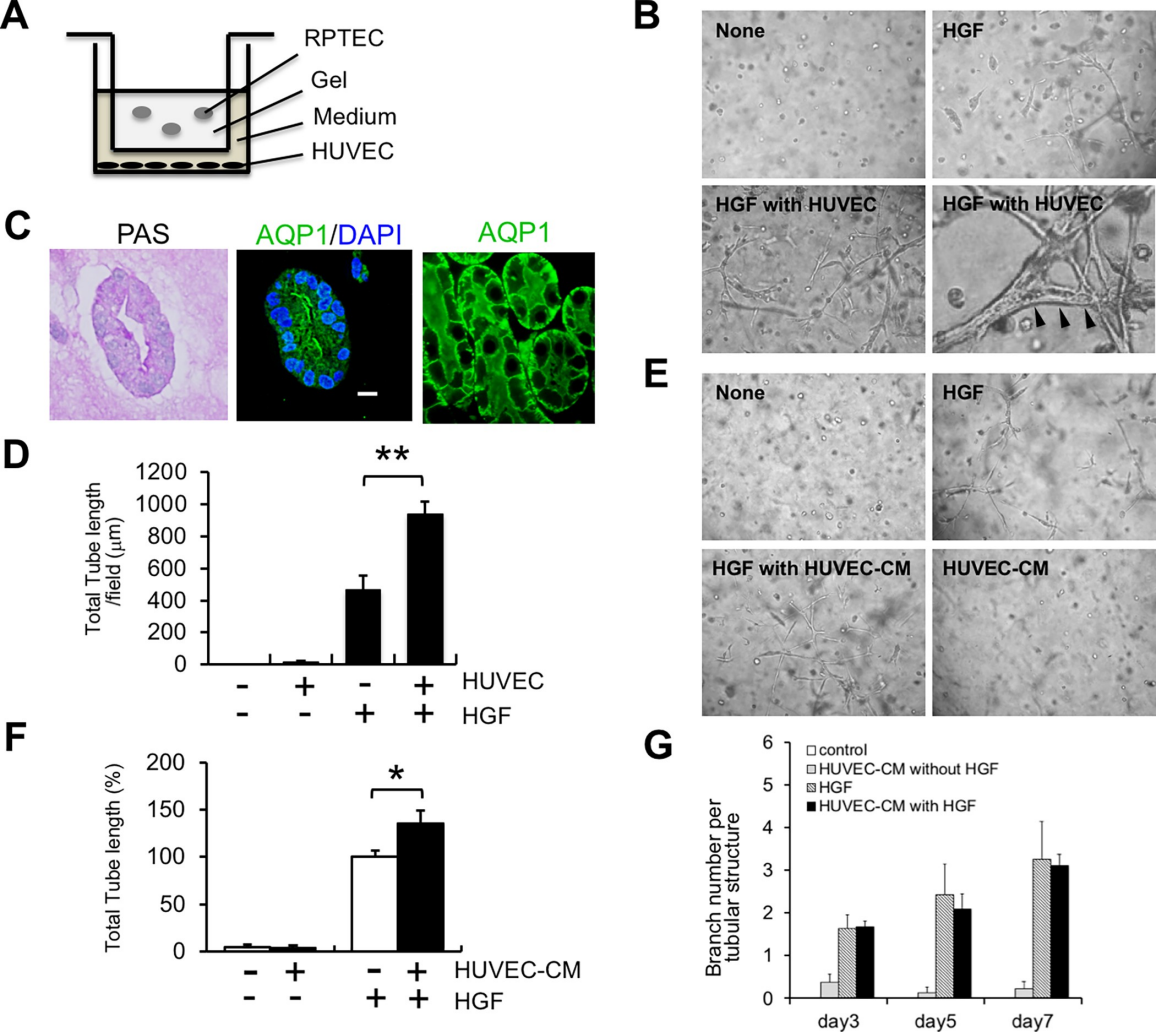
Enhancement of HGF-induced tubule formation by Co-culture with HUVECs or culture in HUVEC-derived conditioned medium (HUVEC-CM). A: illustration of 3-dimensional gel culture system. B: Morphology of RPTECs cultured in gels in the absence or presence of HGF (100 ng/ml) with HUVECs for 5 days (Magnification, ×100 and ×400). Arrowheads indicate the lumen of tubular structures. C: (Left panel) Periodic acid-Schiff (PAS)-stained section of HGF-induced tubular structures after 9 days of culture. Magnification, ×200. (Middle panel) Localization of AQP1 at the apical side of the lumen in HGF-induced tubular structures. AQP1 (green), DAPI (blue). Bar, 10 μm. (Right panel) Localization of AQP1 in renal tubules of rat kidney. AQP1 (green). (Magnification, ×400) D: Quantitative analysis of tubule formation. RPTECs were cultured in gels in the presence of HGF (100 ng/ml) with or without HUVECs for 5 days. Values are the mean ± SE (n = 6). **P < 0.001 E: Morphology of RPTECs cultured in gels in the absence or presence of HGF (100 ng/ml) with or without HUVEC-CM for 5 days (Magnification, ×100). F: Effects of HUVEC-CM on HGF-induced tubule formation. RPTECs were cultured in gels in the presence of HGF (100 ng/ml) with or without HUVEC-CM for 5 days. Values are the mean ± SE (n = 7). *P < 0.05 G: Quantitative analysis of branch number per tubular structure. Values are the mean ± SE (n = 5).

In the present study, we performed an in vitro 3D tubulogenesis assay. Human RPTECs were cultured in gels in the presence of HGF, and morphological changes of RPTECs were examined (Fig 1A). Similar to a previous study[14], RPTECs cultured in gels formed tubular structures in the presence of HGF (Fig 1B, upper right panel), but not in the absence of HGF (Fig 1B, upper left panel). We then examined the phenotype of HGF-induced tubular structures. Periodic acid-Schiff-stained sections revealed the presence of lumens in these tubular structures (Fig 1C, left panel). AQP1, which is localized on both apical and basolateral membranes of proximal tubules in vivo[23] (Fig 1C, right panel), were present at the apical site of the lumen (Fig 1C, middle panel), suggesting that HGF-induced tubular structures are partially morphologically equivalent to renal proximal tubules in vivo.

### Enhancement of HGF-induced tubule formation by Co-culture with HUVECs

We then examined the effect of endothelial cell-derived factors on the tubulogenic action of HGF. RPTECs in gels were co-cultured with HUVECs using a Transwell filter. When co-cultured with HUVECs, HGF-induced tubule formation was extensively enhanced (Fig 1B, lower left panel). Some but not all tubular structures have a lumen (Fig 1B, lower right panel). In contrast, co-culture with HUVECs had no effect on tubule formation in the absence of HGF (data not shown). Quantitatively, the length of tubular structures was significantly increased when co-cultured with HUVECs compared with that without HUVECs (Fig 1D). Consistent with the above results, HGF-induced tubule formation was significantly increased in the presence of HUVEC-CM (Fig 1E and 1F), suggesting the presence of HUVEC-derived soluble factor(s) that enhance HGF-induced tubule formation, although branch number per tubular structure was not significantly different between HGF and HGF with HUVEC-CM (Fig 1G).

### Enhanced phosphorylation of RTKs in HGF-induced tubular structures by HUVEC-CM

To identify the HUVEC-derived factor(s) that enhance HGF-induced tubule formation, we performed the Human Phospho-RTK Array and measured phosphorylation levels of various RTKs in HGF-induced tubular structures cultured with or without HUVEC-CM. Phosphorylation of most RTKs was unchanged or undetectable in HGF-induced tubular structures cultured with HUVEC-CM (Table 1). Among the receptor genes examined, the most upregulated gene in HGF-induced tubular structures cultured with HUVEC-CM was the receptor for GDNF, known as Ret (Table 1, S2 Fig).

**Table 1:** Effect of HUVEC-CM on phosphorylation of Various RTKs in HGF-induced tubular structures.

**Up-regulated**	Mean pixel density		
	HUVEC(-)	HUVEC(+)	Ratio
IGF-I-R	0.00051	0.00131	2.58
c-Ret	0.00166	0.00337	2.04
Insulin-R	0.00384	0.00751	1.95
HGF-R	0.00149	0.00278	1.87
Axl	0.00288	0.00518	1.79
EphA7	0.00209	0.00330	1.58
ROR1	0.00069	0.00109	1.57
ROR2	0.00101	0.00153	1.51
**Down-regulated**	Mean pixel density		
	HUVEC(-)	HUVEC(+)	Ratio
Mer	0.00228	0.00108	0.47
Tie-2	0.00266	0.00104	0.39
EphA6	0.00445	0.00126	0.28
**Unchanged**	Mean pixel density		
	HUVEC(-)	HUVEC(+)	Ratio
EGF-R	0.00607	0.00867	1.43
ErbB2	0.00188	0.00256	1.36
Dtk	0.00349	0.00350	1.00
Tie-1	0.00169	0.00179	1.05
EphA1	0.00090	0.00104	1.33
EphA10	0.00203	0.00206	1.02
EphB8	0.00102	0.00074	0.73
ALK	0.00119	0.00079	0.67
RYK	0.00338	0.00453	1.34
**Undetectable**	Mean pixel density				
	HUVEC(-)	HUVEC(+)	Ratio
ErbB3	N.D.	N.D.	-
ErbB4	N.D.	N.D.	-
FGF-R1	N.D.	N.D.	-
FGF-R2-alpha	N.D.	N.D.	-
FGF-R3	N.D.	N.D.	-
FGF-R4	N.D.	N.D.	-
MSP-R	N.D.	N.D.	-
PDGF-R-alpha	N.D.	N.D.	-
PDGF-R-beta	N.D.	N.D.	-
SCF-R	N.D.	N.D.	-
Flt-3	N.D.	N.D.	-
M-CSF-R	N.D.	N.D.	-
TrkA	N.D.	N.D.	-
TrkB	N.D.	N.D.	-
TrkC	N.D.	N.D.	-
VEGF-R1	N.D.	N.D.	-
VEGF-R2	N.D.	N.D.	-
VEGF-R3	N.D.	N.D.	-
MuSK	N.D.	N.D.	-
EphA2	N.D.	N.D.	-
EphA3	N.D.	N.D.	-
EphA4	N.D.	N.D.	-
EphB1	N.D.	N.D.	-
EphB2	N.D.	N.D.	-
EphB4	N.D.	N.D.	-
EphB6	N.D.	N.D.	-
DDR1	N.D.	N.D.	-
DDR2	N.D.	N.D.	-
EphA5	N.D.	N.D.	-

### Expression of Ret and GFR-alpha in RPTECs

The RTK Ret acts as a common signaling receptor for all GDNF family ligands including GDNF, NRTN, ARTN, and PSPN[24]. The binding specificity of these ligands is determined by GFR-alpha proteins, which have unique binding affinities for each ligand. GDNF, NRTN, ARTN, and PSPN specifically bind to GFR-alpha 1, GFR-alpha 2, GFR-alpha 3, and GFR-alpha 4, respectively, form receptor complexes, and signal through the Ret RTK [25].

**Fig 2:**
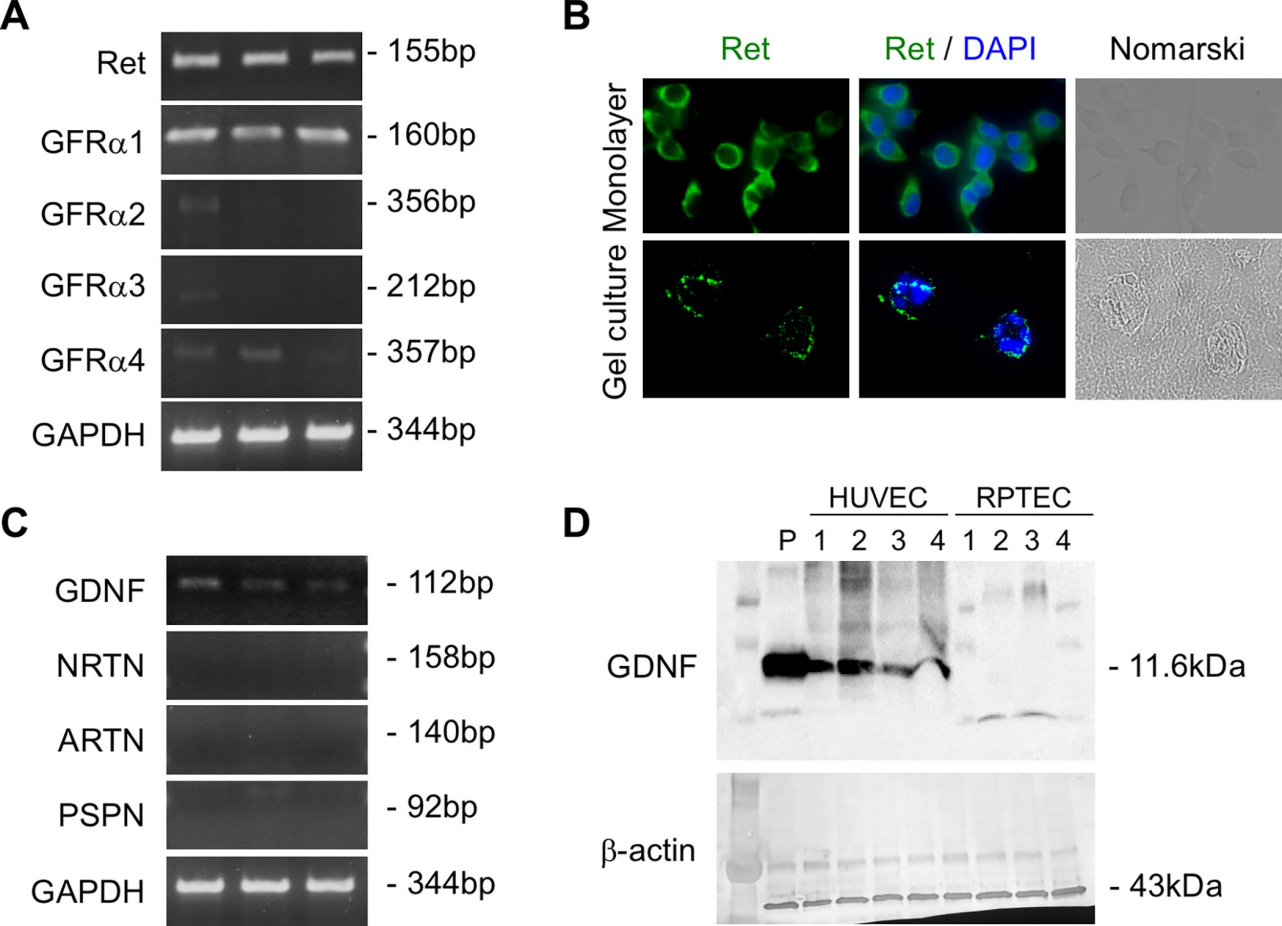
Expression of Ret and GDNF family ligands/receptors in RPTECs and HUVECs. A: The expression of mRNA for Ret, GFR-alpha 1, GFR-alpha 2, GFR-alpha 3, and GFR-alpha 4 in RPTECs cultured in a monolayer were examined with RT-PCR (n = 3). Representative images are shown. B: Expression of Ret in RPTECs cultured in a monolayer for 5 days or cultured in gels for 8 days. Ret (green), DAPI (blue). C: The expression of mRNA for GDNF, NRTN, ARTN, and PSPN in HUVECs cultured in a monolayer was examined with RT-PCR (n = 3). D: Production of GDNF in HUVECs (n = 4) or RPTEC (n = 4) cultured in a monolayer was examined with western blot analysis. P, positive control (rat brain).

We examined the expression of Ret and GFR-alpha proteins in RPTECs with RT-PCR. Expression of Ret and GFR-alpha 1, but not GFR-alpha 2, GFR-alpha 3, or GFR-alpha 4, was detected in RPTECs (Fig 2A). Immunostaining demonstrated that Ret was expressed in RPTECs cultured in a monolayer as well as in the elongated process of RPTEC formed in gels (Fig 2B).

### Production of GDNF family ligands by HUVECs

We next examined the production of GDNF family ligands by HUVECs. RT-PCR demonstrated that HUVECs expressed GDNF, but not NRTN, ARTN, or PSPN (Fig 2C). Western blot analysis showed that GDNF protein was produced by HUVECs, but not by RPTEC (Fig 2D).

### Enhancement of HGF-induced tubule formation by GDNF

**Fig 3:**
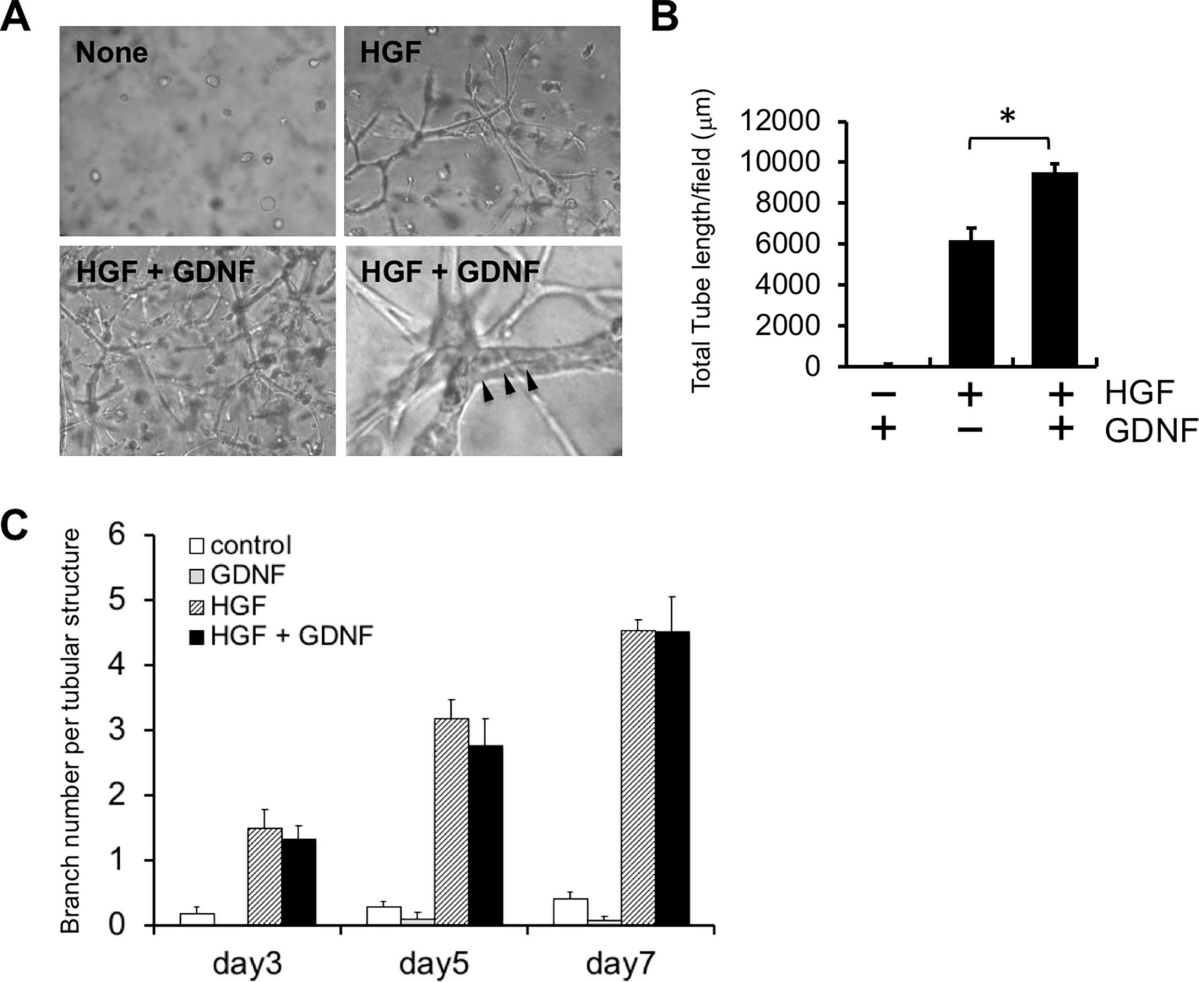
Promotion of HGF-induced tubule formation in the presence of GDNF. A: Morphology of RPTECs cultured in gels in the absence or presence of HGF (50 ng/ml) with GDNF (100 ng/ml) for 5 days (Magnification, ×100 and ×400). Arrowheads indicate the lumen of tubular structures. B: Quantitative analysis of tubule formation. RPTECs were cultured in gels with the indicated factors for 5 days. Values are the mean ± SE (n = 3). *P < 0.05. C: Quantitative analysis of branch number per tubular structure. Values are the mean ± SE (n = 5).

To investigate the effect of GDNF on HGF-induced tubule formation, we cultured RPTECs in gels in the presence of HGF with or without GDNF. HGF-induced tubule formation was enhanced in the presence of GDNF compared to that in the absence of GDNF (Fig 3A). Quantitatively, the length of HGF-induced tubular structures was significantly increased by GDNF compared to that without GDNF (Fig 3B). There was no significant difference between HGF and HGF with GDNF (Fig 3C).

### Phosphorylation of Met by GDNF

**Fig 4:**
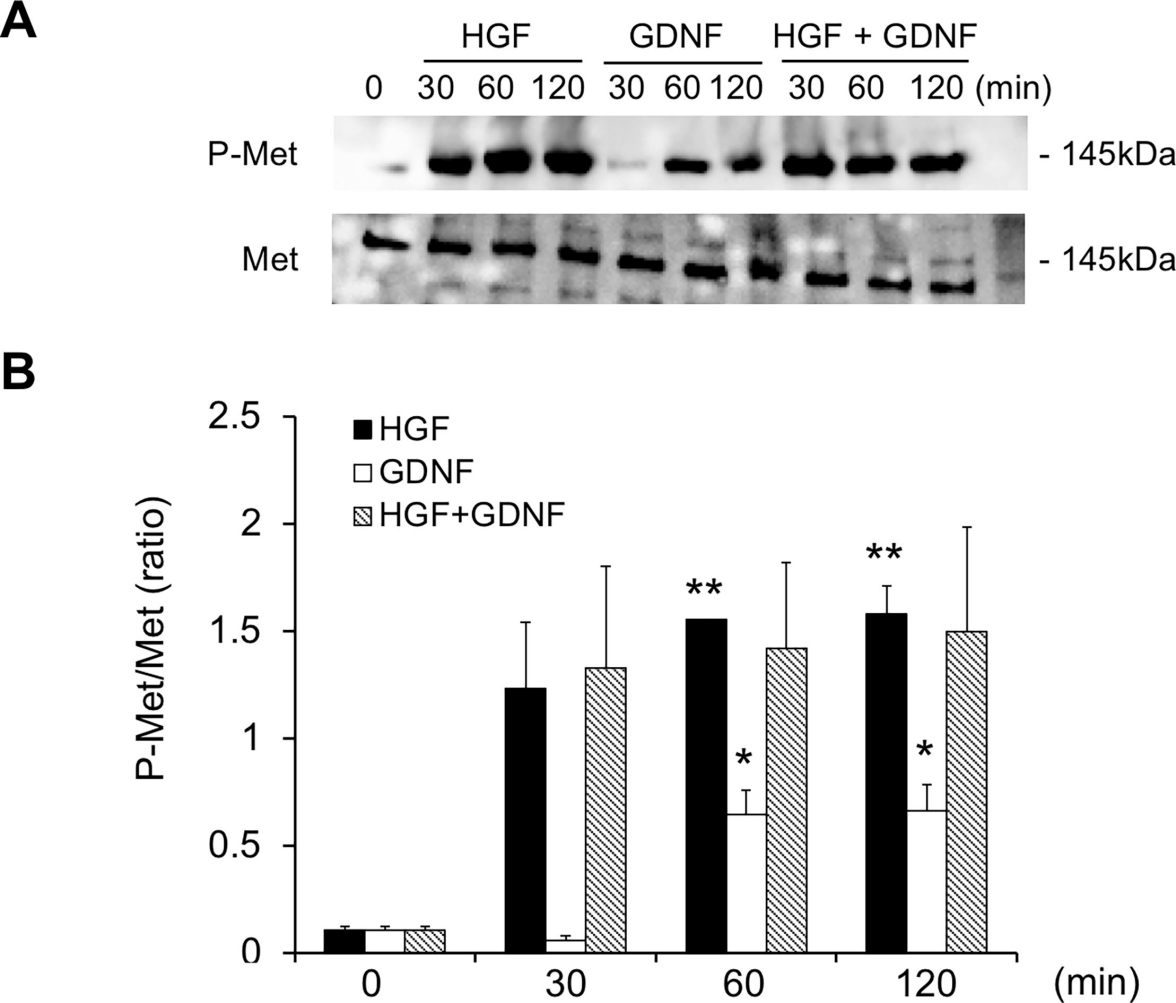
Phosphorylation of Met via GDNF. A: Production of phospho-Met and total Met protein in RPTECs cultured in a monolayer treated with HGF (50 ng/ml), GDNF (50 ng/ml) or HGF plus GDNF was examined with western blot analysis. Representative results of three independent experiments are shown. B: Quantitative analysis of phospho-Met normalized to total Met. Values are the mean ± SE. *P < 0.05, **P < 0.01 vs. 0 min.

GDNF stimulates branching tubulogenesis by increasing phosphorylation of the HGF receptor, Met, in Ret-deficient MDCK cells[26], suggesting the presence of Met-dependent GDNF activity[27]. To test the possibility that GDNF increases HGF-induced tubule formation by enhancing the HGF signaling pathway, we performed western blotting to examine Met phosphorylation in RPTECs cultured in a monolayer. HGF increased phospho-Met in RPTECs 30 min after stimulation and thereafter (Fig 4A). Interestingly, GDNF also increased Met phosphorylation 60 min after stimulation and thereafter in the absence of HGF. Quantitative analysis showed that both HGF and GDNF significantly increased the phosphorylation of Met, but no additive effects were observed (Fig 4B).

## Discussion

Previous reports indicate the importance of crosstalk between tubular epithelial cells and vascular endothelial cells in the kidney. A complex network of communication between microvascular endothelial cells and proximal tubular epithelial cells significantly affects proximal tubular cell function[28, 29]. Tubular epithelial cells regulate transmigration of neutrophils in concert with endothelial cells during inflammation[30]. Tubule formation by MDCK cells is enhanced by culture with supernatant from mouse vascular endothelial cells[31]. Vascular endothelial growth factor produced by RPTECs significantly augments endothelial capillary network formation using a co-culture system[32]. Human proximal tubular cells (HPTCs) stimulate endothelial cells to express a functionally balanced combination of various factors, which in turn improves the performance of HPTCs[33]. In line with these reports, we demonstrated that HGF induced 3D tubular structures that were positive for AQP1 at the apical site of the lumen (Fig 1C), suggesting that these tubular structures are partially morphologically equivalent to renal tubules in vivo. Co-culture with HUVECs or culture with HUVEC-CM significantly enhanced HGF-induced tubulogenesis (Fig 1B and 1E). The Human Phospho-RTK array revealed that phosphorylation of Ret in HGF-induced tubular structures was enhanced by HUVEC-CM (Table 1). HUVECs produced GDNF, one of the ligands for Ret (Fig 2), which significantly enhanced HGF-induced tubulogenesis (Fig 3). Collectively, our data suggest that GDNF is one of the HUVEC-derived factors responsible for this enhancement, although Ret ligands other than GDNF may also enhance HGF-induced tubulogenesis. GDNF may be an important mediator required for epithelial–endothelial interactions during renal tubulogenesis.

GDNF, originally identified as a potent neurotrophic factor for neurons of the central nervous system, is required for normal kidney development. During kidney development, Ret and GFR-alpha 1 are expressed all along the Wolffian duct, while GDNF is expressed only in the metanephric mesenchyme adjacent to the caudal portion of the Wolffian duct[34] [35, 36]. GDNF promotes the budding of the Wolffian duct epithelium to form the primary ureteric bud[37] and also induces elongation and branching of ureteric buds during kidney development[38]. In organ culture system, exogenous GDNF stimulates both branching and proliferation of embryonic kidneys, whereas neutralizing antibodies against GDNF inhibit branching morphogenesis[39]. GDNF-deficient mice display complete renal agenesis[40–42]. Thus, GDNF is essential and indispensable for normal kidney development [43]. The expression of GDNF is not limited to embryonic kidney, and is also expressed in adult human kidney [44] and human cultured mesangial cells[45]. On the other hand, Ret is expressed not only in ureteric buds of developing kidney[46], but also in renal tubules of adult murine kidney[47] and in collecting ducts of adult human kidney[48]. These data suggest that the GDNF-Ret signaling system plays an important role not only in renal organogenesis, but also in tubular cell growth and differentiation in adult kidney.

Renal epithelial tubular cells proliferate actively and differentiate to reconstitute the tubular epithelium during recovery from a variety of insults[49, 50]. Paracrine factors from vascular endothelial cells play an important role in tissue regeneration in various organs[51]. It has been reported that HGF mRNA and HGF protein were expressed in renal interstitial cells, presumably endothelial cells and macrophages after ischemic kidney injury [52]. Met was also activated in renal tubules after acute kidney injury [53]. We demonstrated that Ret was expressed in RPTECs cultured in a monolayer as well as in the cell membrane of elongated process of HGF-induced tubular structures cultured in gel (Fig 2B). The expression of Ret was also observed in the basolateral membrane of proximal tubules in adult human kidney (Nakasatomi M et al. Unpublished observation). Given that in vitro tubulogenesis models partly mimic in vivo tubular regeneration, our data suggest that the GDNF-Ret signaling pathway plays a role in tubular regeneration after injury, although the expression changes of GDNF or Ret during the repair process of the kidney after acute injury remains unclear. GDNF may potentiate the renoprotective action of HGF during tubular repair after injury. Further studies will be needed to clarify this issue.

HGF, acting through the Met receptor, plays an important role in kidney development[15]. HGF stimulates renal tubular epithelial cells to form elongated tubules when grown in 3D gels[7, 13, 54]. In vivo data also indicate a critical role for HGF/Met signaling during tubule formation during kidney development [43, 55] as well as during tubule regeneration after injury [53]. In the present study, we demonstrated that HGF, but not GDNF, induced renal tubulogenesis and that GDNF significantly enhanced HGF-induced tubulogenesis (Fig 3), suggesting that GDNF itself does not have tubulogenic action but can enhance the tubulogenic action of HGF.

How GDNF enhances HGF tubulogenic action remains unknown, but one possibility is that GDNF exerts its effect in a Met-dependent manner [27]. A previous report demonstrated that GDNF stimulates branching tubulogenesis in MDCK cells expressing GFR-alpha 1. GDNF induces Met phosphorylation in several Ret*-*deficient cell types but not in GFR-alpha 1-positive cells, suggesting the presence of GFR-alpha 1-dependent/Ret-independent tubulogenic action of GDNF. Consistent with these data, we demonstrated that GFR-alpha 1 was expressed by RPTEC (Fig 2A). GDNF enhanced Met phosphorylation in the absence of HGF (Fig 4). Although the additive effect of HGF and GDNF on Met phosphorylation was not observed, it is possible that GDNF enhanced HGF-induced tubulogenesis by Met phosphorylation. Our data suggest the presence of a cooperative mechanism between HGF and GDNF via a Met-dependent pathway during renal tubulogenesis.

HUVECs were cultured in HuMedia-EB2 supplemented with HuMedia-EG for 48 h, and HUVEC-derived conditioned medium (HUVEC-CM) was collected. RPTECs were cultured in gels with HuMedia-EB2 supplemented with HuMedia-EG or HUVEC-CM. The Human Phospho-RTK Array Kit was used according to the manufacturer’s instructions. Briefly, after 5 days, proteins were extracted from these gels with lysis buffer, which was a component of this kit, supplemented with 10 μg/ml Aprotinin, 10 μg/ml Leupeptin, and 10 μg/ml Pepstatin. After blocking, arrayed antibody membranes were incubated with 300 μg protein at 4°C overnight. After washing, membranes were incubated with horseradish peroxidase-conjugated antibodies for 2 h at room temperature and reacted with chemiluminescent substrate. The signal was detected with the ImageQuant LAS4000 and quantified using ImageQuant TL. RTKs were classified into four categories: upregulated, downregulated, unchanged, and undetectable.

## Supporting information

S1 Fig List of Primers used in this study. (PDF)

S2 Fig The signals of phosphorylated genes in RPTEC cultured in gels with or without HUVEC-CM. Data of the Human Phospho-RTK Array membrane (Upper panel) and the list of genes corresponding to each spot (Lower panel).
(PDF)

## References

[1] Lubarsky B, Krasnow MA. Tube morphogenesis: making and shaping biological tubes. Cell. 2003;112(1):19–28. . 12526790 10.1016/s0092-8674(02)01283-7

[2] Affolter M, Bellusci S, Itoh N, Shilo B, Thiery JP, Werb Z. Tube or not tube: remodeling epithelial tissues by branching morphogenesis. Developmental cell. 2003;4(1):11–8. . 12530959 10.1016/s1534-5807(02)00410-0

[3] Hogan BL, Kolodziej PA. Organogenesis: molecular mechanisms of tubulogenesis. Nature reviews Genetics. 2002;3(7):513–23. 10.1038/nrg840 . 12094229

[4] Ramasamy SK, Kusumbe AP, Adams RH. Regulation of tissue morphogenesis by endothelial cell-derived signals. Trends in cell biology. 2015;25(3):148–57. 10.1016/j.tcb.2014.11.007 25529933 PMC4943524

[5] Adler M, Ramm S, Hafner M, Muhlich JL, Gottwald EM, Weber E, et al A Quantitative Approach to Screen for Nephrotoxic Compounds In Vitro. J Am Soc Nephrol. 2016;27(4):1015–28. 10.1681/ASN.2015010060 26260164 PMC4814182

[6] Zegers MM, O'Brien LE, Yu W, Datta A, Mostov KE. Epithelial polarity and tubulogenesis in vitro. Trends in cell biology. 2003;13(4):169–76. Epub 2003/04/02. S0962892403000369 [pii]. . 12667754 10.1016/s0962-8924(03)00036-9

[7] Montesano R, Matsumoto K, Nakamura T, Orci L. Identification of a fibroblast-derived epithelial morphogen as hepatocyte growth factor. Cell. 1991;67(5):901–8. . 1835669 10.1016/0092-8674(91)90363-4

[8] Montesano R, Schaller G, Orci L. Induction of epithelial tubular morphogenesis in vitro by fibroblast-derived soluble factors. Cell. 1991;66(4):697–711. . 1878968 10.1016/0092-8674(91)90115-f

[9] Cantley LG, Barros EJ, Gandhi M, Rauchman M, Nigam SK. Regulation of mitogenesis, motogenesis, and tubulogenesis by hepatocyte growth factor in renal collecting duct cells. Am J Physiol. 1994;267(2 Pt 2):F271–80. 10.1152/ajprenal.1994.267.2.F271 . 8067388

[10] Barros EJ, Santos OF, Matsumoto K, Nakamura T, Nigam SK. Differential tubulogenic and branching morphogenetic activities of growth factors: implications for epithelial tissue development. Proc Natl Acad Sci U S A. 1995;92(10):4412–6. . 7753820 10.1073/pnas.92.10.4412 PMC41954

[11] Derman MP, Cunha MJ, Barros EJ, Nigam SK, Cantley LG. HGF-mediated chemotaxis and tubulogenesis require activation of the phosphatidylinositol 3-kinase. Am J Physiol. 1995;268(6 Pt 2):F1211–7. 10.1152/ajprenal.1995.268.6.F1211 . 7611461

[12] Sakurai H, Barros EJ, Tsukamoto T, Barasch J, Nigam SK. An in vitro tubulogenesis system using cell lines derived from the embryonic kidney shows dependence on multiple soluble growth factors. Proc Natl Acad Sci U S A. 1997;94(12):6279–84. . 9177208 10.1073/pnas.94.12.6279 PMC21040

[13] Bowes RC 3rd, Lightfoot RT, Van De Water B, Stevens JL. Hepatocyte growth factor induces tubulogenesis of primary renal proximal tubular epithelial cells. J Cell Physiol. 1999;180(1):81–90. 10.1002/(SICI)1097-4652(199907)180:1<81::AID-JCP9>3.0.CO;2-J . 10362020

[14] Miya M, Maeshima A, Mishima K, Sakurai N, Ikeuchi H, Kuroiwa T, et al Enhancement of in vitro human tubulogenesis by endothelial cell-derived factors: implications for in vivo tubular regeneration after injury. Am J Physiol Renal Physiol. 2011;301(2):F387–95. 10.1152/ajprenal.00619.2010 . 21561997

[15] Liu Y. Hepatocyte growth factor and the kidney. Curr Opin Nephrol Hypertens. 2002;11(1):23–30. . 11753083 10.1097/00041552-200201000-00004

[16] Maeshima A, Maeshima K, Nojima Y, Kojima I. Involvement of Pax-2 in the action of activin A on tubular cell regeneration. J Am Soc Nephrol. 2002;13(12):2850–9. . 12444203 10.1097/01.asn.0000035086.93977.e9

[17] Spinnler K, Kohn FM, Schwarzer U, Mayerhofer A. Glial cell line-derived neurotrophic factor is constitutively produced by human testicular peritubular cells and may contribute to the spermatogonial stem cell niche in man. Hum Reprod. 2010;25(9):2181–7. 10.1093/humrep/deq170 . 20601681

[18] Hishiki T, Nimura Y, Isogai E, Kondo K, Ichimiya S, Nakamura Y, et al Glial cell line-derived neurotrophic factor/neurturin-induced differentiation and its enhancement by retinoic acid in primary human neuroblastomas expressing c-Ret, GFR alpha-1, and GFR alpha-2. Cancer Res. 1998;58(10):2158–65. . 9605760

[19] Kang J, Perry JK, Pandey V, Fielder GC, Mei B, Qian PX, et al Artemin is oncogenic for human mammary carcinoma cells. Oncogene. 2009;28(19):2034–45. 10.1038/onc.2009.66 . 19363524

[20] Baba T, Sakamoto Y, Kasamatsu A, Minakawa Y, Yokota S, Higo M, et al Persephin: A potential key component in human oral cancer progression through the RET receptor tyrosine kinase-mitogen-activated protein kinase signaling pathway. Molecular carcinogenesis. 2015;54(8):608–17. 10.1002/mc.22127 . 24375483

[21] Sasaki H, Shimizu S, Tani Y, Maekawa M, Okuda K, Yokota K, et al RET expression and detection of KIF5B/RET gene rearrangements in Japanese lung cancer. Cancer medicine. 2012;1(1):68–75. 10.1002/cam4.13 23342255 PMC3544433

[22] Murota H, Izumi M, Abd El-Latif MI, Nishioka M, Terao M, Tani M, et al Artemin causes hypersensitivity to warm sensation, mimicking warmth-provoked pruritus in atopic dermatitis. J Allergy Clin Immunol. 2012;130(3):671–82 e4. 10.1016/j.jaci.2012.05.027 . 22770266

[23] Agre P. Homer W. Smith award lecture. Aquaporin water channels in kidney. J Am Soc Nephrol. 2000;11(4):764–77. . 10752537 10.1681/ASN.V114764

[24] Takahashi M. The GDNF/RET signaling pathway and human diseases. Cytokine Growth Factor Rev. 2001;12(4):361–73. . 11544105 10.1016/s1359-6101(01)00012-0

[25] Airaksinen MS, Saarma M. The GDNF family: signalling, biological functions and therapeutic value. Nature reviews Neuroscience. 2002;3(5):383–94. 10.1038/nrn812 . 11988777

[26] Popsueva A, Poteryaev D, Arighi E, Meng X, Angers-Loustau A, Kaplan D, et al GDNF promotes tubulogenesis of GFRalpha1-expressing MDCK cells by Src-mediated phosphorylation of Met receptor tyrosine kinase. J Cell Biol. 2003;161(1):119–29. 10.1083/jcb.200212174 12682085 PMC2172872

[27] Sariola H, Saarma M. Novel functions and signalling pathways for GDNF. Journal of cell science. 2003;116(Pt 19):3855–62. 10.1242/jcs.00786 . 12953054

[28] Aydin S, Signorelli S, Lechleitner T, Joannidis M, Pleban C, Perco P, et al Influence of microvascular endothelial cells on transcriptional regulation of proximal tubular epithelial cells. Am J Physiol Cell Physiol. 2008;294(2):C543–54. 10.1152/ajpcell.00307.2007 . 18057119

[29] Linas SL, Repine JE. Endothelial cells regulate proximal tubule epithelial cell sodium transport. Kidney Int. 1999;55(4):1251–8. 10.1046/j.1523-1755.1999.00360.x . 10200988

[30] Bijuklic K, Jennings P, Kountchev J, Hasslacher J, Aydin S, Sturn D, et al Migration of leukocytes across an endothelium-epithelium bilayer as a model of renal interstitial inflammation. Am J Physiol Cell Physiol. 2007;293(1):C486–92. 10.1152/ajpcell.00419.2006 . 17428840

[31] Machiguchi T, Nakamura T. Cellular interactions via conditioned media induce in vivo nephron generation from tubular epithelial cells or mesenchymal stem cells. Biochem Biophys Res Commun. 2013;435(3):327–33. 10.1016/j.bbrc.2013.04.050 . 23618853

[32] Kim BS, Chen J, Weinstein T, Noiri E, Goligorsky MS. VEGF expression in hypoxia and hyperglycemia: reciprocal effect on branching angiogenesis in epithelial-endothelial co-cultures. J Am Soc Nephrol. 2002;13(8):2027–36. . 12138133 10.1097/01.asn.0000024436.00520.d8

[33] Tasnim F, Zink D. Cross talk between primary human renal tubular cells and endothelial cells in cocultures. Am J Physiol Renal Physiol. 2012;302(8):F1055–62. 10.1152/ajprenal.00621.2011 . 22319059

[34] Pachnis V, Mankoo B, Costantini F. Expression of the c-ret proto-oncogene during mouse embryogenesis. Development. 1993;119(4):1005–17. . 8306871 10.1242/dev.119.4.1005

[35] Hellmich HL, Kos L, Cho ES, Mahon KA, Zimmer A. Embryonic expression of glial cell-line derived neurotrophic factor (GDNF) suggests multiple developmental roles in neural differentiation and epithelial-mesenchymal interactions. Mechanisms of development. 1996;54(1):95–105. . 8808409 10.1016/0925-4773(95)00464-5

[36] Sainio K, Suvanto P, Davies J, Wartiovaara J, Wartiovaara K, Saarma M, et al Glial-cell-line-derived neurotrophic factor is required for bud initiation from ureteric epithelium. Development. 1997;124(20):4077–87. . 9374404 10.1242/dev.124.20.4077

[37] Costantini F, Shakya R. GDNF/Ret signaling and the development of the kidney. BioEssays: news and reviews in molecular, cellular and developmental biology. 2006;28(2):117–27. 10.1002/bies.20357 . 16435290

[38] Dressler GR. The cellular basis of kidney development. Annu Rev Cell Dev Biol. 2006;22:509–29. 10.1146/annurev.cellbio.22.010305.104340 . 16822174

[39] Vega QC, Worby CA, Lechner MS, Dixon JE, Dressler GR. Glial cell line-derived neurotrophic factor activates the receptor tyrosine kinase RET and promotes kidney morphogenesis. Proc Natl Acad Sci U S A. 1996;93(20):10657–61. . 8855235 10.1073/pnas.93.20.10657 PMC38210

[40] Pichel JG, Shen L, Sheng HZ, Granholm AC, Drago J, Grinberg A, et al Defects in enteric innervation and kidney development in mice lacking GDNF. Nature. 1996;382(6586):73–6. 10.1038/382073a0 . 8657307

[41] Sanchez MP, Silos-Santiago I, Frisen J, He B, Lira SA, Barbacid M. Renal agenesis and the absence of enteric neurons in mice lacking GDNF. Nature. 1996;382(6586):70–3. 10.1038/382070a0 . 8657306

[42] Moore MW, Klein RD, Farinas I, Sauer H, Armanini M, Phillips H, et al Renal and neuronal abnormalities in mice lacking GDNF. Nature. 1996;382(6586):76–9. 10.1038/382076a0 . 8657308

[43] Costantini F, Kopan R. Patterning a complex organ: branching morphogenesis and nephron segmentation in kidney development. Developmental cell. 2010;18(5):698–712. 10.1016/j.devcel.2010.04.008 20493806 PMC2883254

[44] Yamamoto M, Sobue G, Yamamoto K, Terao S, Mitsuma T. Expression of mRNAs for neurotrophic factors (NGF, BDNF, NT-3, and GDNF) and their receptors (p75NGFR, trkA, trkB, and trkC) in the adult human peripheral nervous system and nonneural tissues. Neurochem Res. 1996;21(8):929–38. . 8895847 10.1007/BF02532343

[45] Orth SR, Ritz E, Suter-Crazzolara C. Glial cell line-derived neurotrophic factor (GDNF) is expressed in the human kidney and is a growth factor for human mesangial cells. Nephrol Dial Transplant. 2000;15(5):589–95. . 10809797 10.1093/ndt/15.5.589

[46] Tsuzuki T, Takahashi M, Asai N, Iwashita T, Matsuyama M, Asai J. Spatial and temporal expression of the ret proto-oncogene product in embryonic, infant and adult rat tissues. Oncogene. 1995;10(1):191–8. . 7824273

[47] Chan SY, Lee DC. Sex difference in immunostaining of RET in the adult mouse kidney. Oncogene. 1998;17(5):661–6. 10.1038/sj.onc.1201970 . 9704933

[48] Lee DC, Chan KW, Chan SY. RET receptor tyrosine kinase isoforms in kidney function and disease. Oncogene. 2002;21(36):5582–92. 10.1038/sj.onc.1205741 . 12165857

[49] Little MH, Kairath P. Does Renal Repair Recapitulate Kidney Development? J Am Soc Nephrol. 2016 10.1681/ASN.2016070748 . 27798243 PMC5198297

[50] Maeshima A, Takahashi S, Nakasatomi M, Nojima Y. Diverse Cell Populations Involved in Regeneration of Renal Tubular Epithelium following Acute Kidney Injury. Stem cells international. 2015;2015:964849 10.1155/2015/964849 26089922 PMC4452180

[51] Rafii S, Butler JM, Ding BS. Angiocrine functions of organ-specific endothelial cells. Nature. 2016;529(7586):316–25. 10.1038/nature17040 26791722 PMC4878406

[52] Igawa T, Matsumoto K, Kanda S, Saito Y, Nakamura T. Hepatocyte growth factor may function as a renotropic factor for regeneration in rats with acute renal injury. Am J Physiol. 1993;265(1 Pt 2):F61–9. 10.1152/ajprenal.1993.265.1.F61 . 8342615

[53] Zhou D, Tan RJ, Lin L, Zhou L, Liu Y. Activation of hepatocyte growth factor receptor, c-met, in renal tubules is required for renoprotection after acute kidney injury. Kidney Int. 2013;84(3):509–20. 10.1038/ki.2013.102 23715119 PMC3758808

[54] Maeshima A, Zhang YQ, Furukawa M, Naruse T, Kojima I. Hepatocyte growth factor induces branching tubulogenesis in MDCK cells by modulating the activin-follistatin system. Kidney Int. 2000;58(4):1511–22. 10.1046/j.1523-1755.2000.00313.x . 11012886

[55] Rosario M, Birchmeier W. How to make tubes: signaling by the Met receptor tyrosine kinase. Trends in cell biology. 2003;13(6):328–35. Epub 2003/06/07. S0962892403001041 [pii]. . 12791299 10.1016/s0962-8924(03)00104-1

