# Supplementary material for: Enhancement of HGF-induced tubulogenesis by endothelial cell-derived GDNF
Source: PLoS One. 2019 Mar 7;14(3):e0212991. doi: 10.1371/journal.pone.0212991 (PMC6405134; doi:10.1371/journal.pone.0212991)
Supplement: S1 Fig — (PDF) [file pone.0212991.s001.pdf]

Supplemental Figure 1

Primers used in this study

GDNF	Forward	5'-TGGCAGTGCTTCCTAGAAGAG-3'
	Reverse	5'-AAGACACAACCCCGGTTTTTG-3'
Neurturin	Forward	5'-ACGGTGCTGTTCCGCTACTG-3'
	Reverse	5'-AAGGACACCTCGTCCTCGTAG-3'
Artemin	Forward	5'-TGCTGAGCAGCGTCGCAGAG-3'
	Reverse	5'-GCTCTTCCACTGCACCAGCG-3'
Persephin	Forward	5'-TCTGAACAGGTGGCAAAGG-3'
	Reverse	5'-AGCTGGCATGGACCAGAC-3'
Ret	Forward	5'-ACAGGGGATGCAGTATCTGG-3'
	Reverse	5'-CCTGGCTCCTCTTCACGTAG-3'
GFRα1	Forward	5'-TGGAGGATTCCCCATATGAA-3'
	Reverse	5'-TTCTTGCAAATGTCGTCGAG-3'
GFRα2	Forward	5'-GCTGGCATGATTGGGTTTGA-3'
	Reverse	5'-TTGGAGTTGTTGGCCTTCAG-3'
GFRα3	Forward	5'-GTGTGAAATGCTGGAAGGGT-3'
	Reverse	5'-TCAGGAGCAGAATCAAGGGA-3'
GFRα4	Forward	5'-CTCTCCATACTTCCTGTCCT-3'
	Reverse	5'-CTACAAAAGTGACCCTCTCC-3'
GAPDH	Forward	5'-TTGGTATCGTGGAAGGACTCA-3'
	Reverse	5'-GACCACCTGGTGCTCAGTGTA-3'
