# Supplementary material for: Enhancement of HGF-induced tubulogenesis by endothelial cell-derived GDNF
Source: PLoS One. 2019 Mar 7;14(3):e0212991. doi: 10.1371/journal.pone.0212991 (PMC6405134; doi:10.1371/journal.pone.0212991)
Supplement: S2 Fig — Data of the Human Phospho-RTK Array membrane (Upper panel) and the list of genes corresponding to each spot (Lower panel). (PDF) [file pone.0212991.s002.pdf]

HUVEC-CM (-)

HUVEC-CM (+)

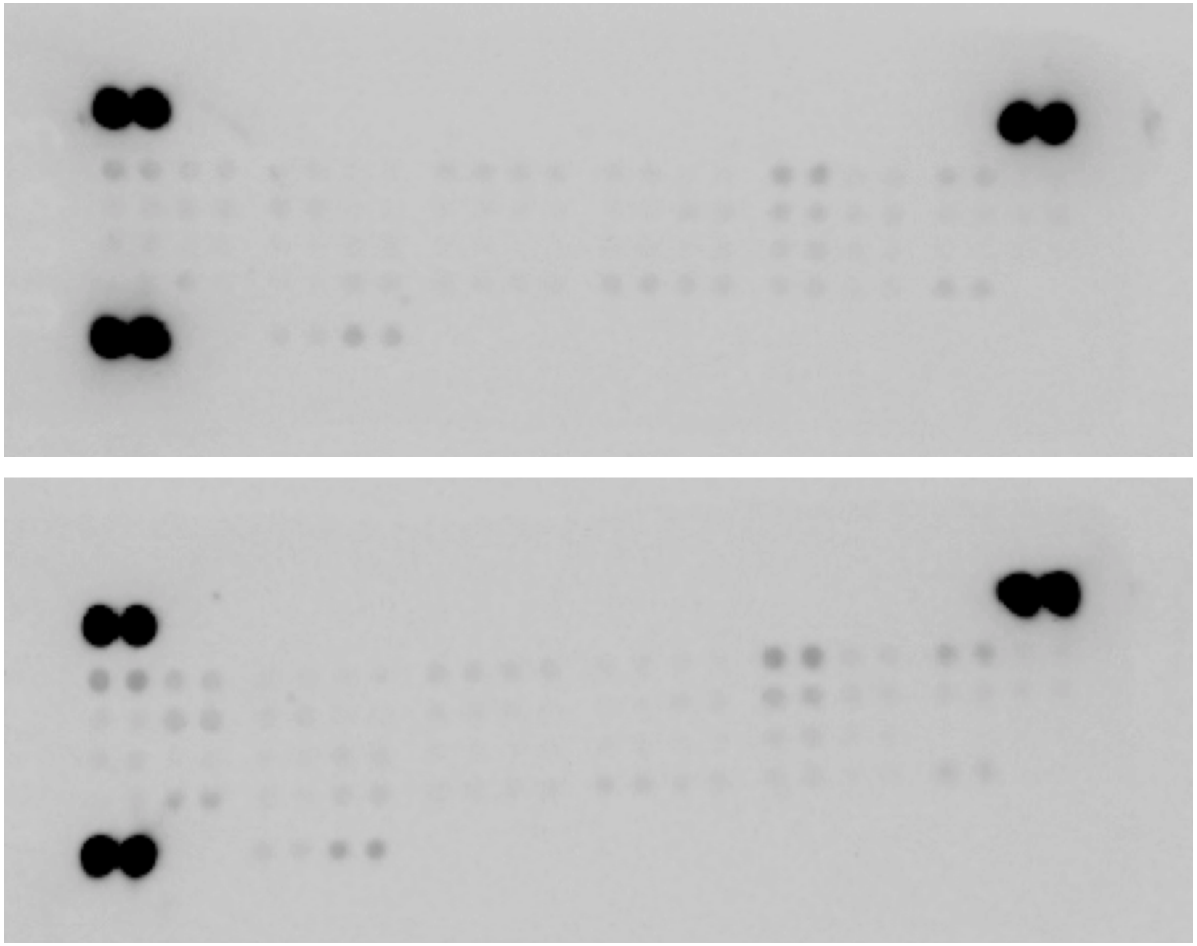

Positive control											Positive control
EGF-R	ErbB2	ErbB3	ErbB4	FGF-R1	FGF-R2α	FGF-R3	FGF-R4	Insulin-R	IGF-I-R	Axl	Dtk
Mer	HGF-R	MSP-R	PDGF-Rα	PDGF-Rβ	SCF-R	Flt-3	M-CSF-R	c-Ret	ROR1	ROR2	Tie-1
Tie-2	TrkA	TrkB	TrkC	VEGF-R1	VEGF-R2	VEGF-R3	MuSK	EphA1	EphA2	EphA3	EphA4
EphA6	EphA7	EphB1	EphB2	EphB4	EphB6	ALK	DDR1	DDR2	EphA5	EphA10	
Positive control		EphB8	RYK								Negative control
